# Techniques and Technologies to Improve Vein Graft Patency in Coronary Surgery

**DOI:** 10.3390/medsci12010006

**Published:** 2024-01-11

**Authors:** Marco Gemelli, Mariangela Addonizio, Veronica Geatti, Michele Gallo, Lauren K. Dixon, Mark S. Slaughter, Gino Gerosa

**Affiliations:** 1 Cardiac Surgery Unit, Department of Cardiac, Thoracic, Vascular Sciences and Public Health, University of Padua, 35128 Padova, Italy; mariangela.addonizio@studenti.unipd.it (M.A.); veronica.geatti@studenti.unipd.it (V.G.); gino.gerosa@unipd.it (G.G.); 2 Department of Cardiothoracic Surgery, University of Louisville, Louisville, KY 40292, USA; michelegallo@hotmail.co.uk (M.G.); mark.slaughter@louisville.edu (M.S.S.); 3 Clinical Effectiveness Unit, The Royal College of Surgeons of England, London WC2A 3PE, UK; lauren.dixon@bristol.ac.uk

**Keywords:** CABG, vein graft, graft patency, long-term outcomes, vein graft failure

## Abstract

Vein grafts are the most used conduits in coronary artery bypass grafting (CABG), even though many studies have suggested their lower patency compared to arterial alternatives. We have reviewed the techniques and technologies that have been investigated over the years with the aim of improving the quality of these conduits. We found that preoperative and postoperative optimal medical therapy and no-touch harvesting techniques have the strongest evidence for optimizing vein graft patency. On the other hand, the use of venous external support, endoscopic harvesting, vein preservation solution and anastomosis, and graft configuration need further investigation. We have also analyzed strategies to treat vein graft failure: when feasible, re-doing the CABG and native vessel primary coronary intervention (PCI) are the best options, followed by percutaneous procedures targeting the failed grafts.

## 1. Introduction

Coronary artery bypass grafting (CABG) is the most common cardiac surgery and aims to treat coronary artery disease. It consists of bypassing coronary stenosis with autologous grafts, and both arteries and veins are used. The most frequently used conduits in this setting are the left internal mammary artery (LIMA), which usually is grafted to the left anterior descending (LAD) artery, and saphenous vein grafts (SVGs) anastomosed to the other targets [1,2,3]. Other arterial conduits that can be used include the right internal mammary artery (RIMA), the radial artery (RA), and the gastroepiploic artery (GEA). Despite many studies having shown superiority in terms of survival and patency for multiple or total arterial revascularization, SVGs are the most commonly used conduits after the LIMA, and >80% of CABG in the United States comprise vein grafts [4]. SVGs are comparatively easy to harvest, readily available in great lengths to construct more than one graft, and there are no specific contraindications to use them, unlike for RITA and RA, but many studies showed a high rate of early and late occlusion [5]. Recent data suggests that 10 to 15% of the vein conduits are occluded at 1 year, and the 10-year patency is around 60%; conversely, the 10-year patency of both internal mammary and radial arteries are reported to be over 90% [6,7,8,9,10,11]. The numerous and varying mechanisms underlying the low patency of vein grafts have been deeply studied. Different techniques and technologies have been evaluated to prevent these events and to improve vein graft patency and the aim of this review is to assess the current available evidence [12].

## 2. Pathophysiology of Vein Graft Failure

The very early failure, which occurs within the first weeks after the operation, is mainly driven by technical factors and thrombotic events, while intimal hyperplasia is the main responsible factor for the failure after the first month, and atherosclerosis, which develops from the latter, is the main cause of late failure [13,14]. One of the main causes of early graft failure is acute thrombosis, which is mainly due to the harvesting of the graft. The surgical injury causes wall stress with an increase in shear stress and hypoxia due to the section of vasa vasorum, which takes the vein graft to high oxidative stress [14,15,16,17]. These provoke endothelium dysfunction and the loss of balance between antithrombotic and prothrombotic factors, with a switch to a prothrombotic state. Studies showed that the surgical harvesting of vein grafts provokes a reduction of nitric oxide (NO), prostacyclin (PGI_2_), thrombomodulin, and heparin-like substances, while a marked increase in the expression of adhesion molecules, sensitivity to vasoconstrictors, and promotion of coagulation cascade with platelet activation and generation of different pro-coagulation factors [18,19,20]. The interplay between all these factors provokes acute graft occlusive thrombosis, which causes around 10% of the vein graft failure [16]. Technical factors also play an important role in early graft failure. Not only the harvesting techniques but also the quality of the anastomoses and the graft-target vessel size mismatch are responsible for this phenomenon. On the other hand, intimal hyperplasia is the main cause of vein graft failure in the first year after CABG and is characterized by the migration and proliferation of vascular smooth muscle cells from the media into the intima, where they proliferate due to the production of fibroblastic factors. This proliferation causes the thickening of the intima and the following stenosis of the graft [21,22,23]. There is a link between the vascular smooth muscular cells and the endothelial cells and their interaction controls cell proliferation, vascular tone, and response to inflammation [24]. In a normal state, the vascular smooth muscular cells are kept quiescent via molecules like nitric oxide. This avoids the switching of vascular smooth muscular cells to a more proliferative phenotype and regulates the tone of the vessel, moderating the balance between vasoconstriction and vasodilation. The harvesting procedure causes local inflammation and endothelial injury, and this provoked an alteration of the relation between vascular smooth muscular and endothelial cells. The production of specific cytokines and mediators and the interaction between vascular smooth muscular and endothelial cells cause the switching of the first to a proliferative condition, which cause the thickening of the intima called intimal hyperplasia. Graft intimal hyperplasia is mainly seen at the anastomotic sites in the first months, and this is probably due to the higher wall stress on this site due to the higher blood pressure and the influence of the peripheral run-off on the wall stress [25]. The complete process of development of intimal hyperplasia is still not fully understood, but many studies showed a correlation between this and first-year vein graft failure [26]. Finally, late vein graft failure is mainly due to atherosclerosis, which is, in part, a consequence of intimal hyperplasia and progresses much more rapidly in these conduits than in native arteries [27]. The vein graft atherosclerotic plaque is more populated with foam cells and inflammatory cells than in the native arteries, and this is probably due to the higher degree of inflammation caused by the trauma of the harvesting. Vein graft atherosclerosis is usually diffuse and concentric. Different from the classic atherosclerotic plaque, it is very friable with an almost absent fibrous cap and few calcifications. This makes it more prone to rupture [14,28,29,30].

## 3. Patient Comorbidities

Risk factors for atherosclerosis play an important role not only before surgery in the development of coronary artery disease but also in the vein graft long-term patency rate. Smoking and dyslipidemia have been found as the two main atherosclerotic risk factors for vein graft failure. Studies have underlined that inflammatory status is one of the main characteristics of intimal hyperplasia, and atherosclerosis and smoking are major contributors to this [22]. Smoking has been found to be an independent risk predictor of mortality and the need for repeat revascularization in patients who underwent venous graft coronary surgery. Furthermore, a correlation between smoking status and vein graft patency has been demonstrated [16,31,32,33]. Therefore, smoking cessation should be a priority after a CABG procedure, particularly if vein grafts are used. High cholesterol and triglyceride levels have also been associated with vein graft disease and, subsequently, failure. Moreover, studies have shown a strong association between long-term mortality and high cholesterol levels after coronary surgery, and aggressive treatment of dyslipidemia should be prioritized to improve long-term outcomes [34,35,36].

## 4. Surgical Techniques

Different surgical strategies have been used over the years to try to mitigate the phenomenon of early vein graft failure, and those with the strongest evidence are discussed here. 

### 4.1. No-Touch Harvesting Technique

Traditionally, the vein graft was harvested in a skeletonized fashion, avoiding excising all the surrounding tissue, which was left to favor wound healing. On the other hand, the “no-touch” harvesting technique consists of harvesting the SVG with its pedicle, avoiding any pressurized distension, and minimizing trauma to the vessel. Similar to the pedicled LIMA harvesting technique, “no-touch” vein harvesting has shown better results than its skeletonized comparator in many studies. Currently, the 2018 European Society of Cardiology/European Association for Cardio-Thoracic Surgery Guidelines on Myocardial Revascularization recommends the “no-touch” technique as a class IIa level of evidence (LoE) B recommendation when an open harvesting technique is performed [5,37]. Randomized controlled trials have compared the outcome of patients undergoing “no-touch” harvesting of SVG (NT-SVG) rather than conventional skeletonized one (C-SVG). Souza et al. in 2011 reported significantly better conduit patency of the “no-touch” veins at 18 months; these results were subsequently confirmed at 8 and 16 years. Furthermore, the patency of NT-SVG was found to be comparable to that of the LIMA [38,39,40]. In a more recent randomized trial, the NT-SVG was found to be similar to the radial artery in terms of patency at an 8-year follow-up, making it a strong alternative in clinical scenarios that are not ideal for radial grafts [41]. SUPERIOR-SVG was the first multicentric RCT comparing NT-SVG versus C-SVG and demonstrated that the former was not associated with a statistically significant improvement in patency at 1-year follow-up, yet the rate of significant graft stenosis or occlusion in the NT-SVG group was half (7.8%) of that in the control group (15%). On the other hand, the rate of leg wound infection was higher in the NT-SVG group (23.3% vs. 9.5%) [42]. Tian et al. recently published the results of a large multicentric trial including 2655 patients randomly assigned to NT-SVG and C-SVG, and the former resulted in a significant reduction in vein graft occlusion and improvement in patient prognosis [43]. There are currently two ongoing trials assessing outcomes of the “no-touch” technique: the IMPROVE-CABG trial, which aims to assess 5-year angiographic patency in 100 patients randomly assigned to NT-SVG or C-SVG, and the SWEDEGRAFT trial, which has recruited 902 patients to assess 2-year CCTA patency [44,45]. Gaudino et al., in a recent network meta-analysis comparing all the non-LIMA conduits used in CABG, showed that NT-SVG has better patency compared with C-SVG and is not inferior to the radial [46]. In conclusion, data in favor of “no-touch” vein harvesting seem to be quite strong in terms of better patency, and this technique is also recommended in the guidelines. Nevertheless, the risk of increased leg wound infection must be taken into consideration.

### 4.2. Venous External Support

In 1963, Parsonnet et al. had the idea to reduce the mismatch between the vein and the coronary artery using external support for the graft, with the aim to reduce the dilatation of the vein when subjected to high arterial pressure [21,47]. A jugular vein was covered with a monofilament knitted graft, and this was implanted in a dog. At an angiographic follow-up, they showed that the dilation of the stented graft was limited compared to a control. In the following years, many benefits, such as reducing intimal hyperplasia, reducing dilatation of the graft, and increasing the patency, have been demonstrated in animal studies [48,49,50]. After many disappointing results in humans with different types of external stents, the venous external support (VEST) external stent (Vascular Graft Solutions, Tel Aviv, Israel) emerged [51,52,53,54]. It is a cobalt-chrome braid with axial plasticity, which allows elongation and radial elasticity, resulting in kink and crush resistance. After initial promising studies in animals, Taggart and colleagues began the first-in-human trial [55,56]. The VEST I trial enrolled 30 patients with multi-vessel disease, and each patient received, in addition to a LIMA on LAD graft, one external stent to a single SVG (VEST group) randomly allocated to the right or left coronary territories and one or more non-stented veins to the other territories, which served as controls. At the 1-year angiographic follow-up, the SVG failure did not differ between the groups, but the intimal hyperplasia was significantly reduced in the VEST group [56]. Furthermore, there was some evidence that ligation of side branches with sutures rather than metallic clips resulted in a more uniform SVG lumen diameter in the VEST group. This was confirmed in a post-market sub-analysis, which demonstrated that avoidance of both metallic clips to ligate side branches and fixation of stents to the anastomoses marked a significant improvement in the patency of stented SVG to the right coronary territory [57]. The VEST III trial was the first multicenter randomized trial that aimed to validate the VEST I results in a larger cohort (184 patients). At a 2-year follow-up, VEST-supported veins had a lower degree of intimal hyperplasia and an improved Fitzgibbon patency scale (FPS), which is a classification to assess graft lumen non-uniformity. No difference in terms of graft failure was found [58]. Similarly, the VEST Pivotal trial was the first North American multicenter randomized trial to assess angiographic and clinical outcomes of VEST-supported veins in CABG. The primary outcome of this study was the degree of intimal hyperplasia at 12 months, and the results were similar to the VEST III, but the difference in intimal hyperplasia area did not reach a statistical significance [59].

Goldstein et al., in a sub-analysis of the VEST Pivotal data, showed that intimal hyperplasia is associated with clinical measures of SVG disease, such as poorer lumen uniformity, higher level of stenosis, and worse graft perfusion. They have also found that an increased burden of SVG disease is associated with a higher rate of MACCE, including the need for revascularization, at 3 years [26]. Despite this, neither a single RCT nor a recently published meta-analysis performed by our group has shown an advantage in terms of the rate of graft failure of veins treated with VEST [21]. We showed that, despite a clear advantage of VEST-supported veins in terms of intimal hyperplasia, graft non-uniformity, and graft ectasia, no difference has been found in terms of graft failure. We speculated that while the available follow-up could perhaps be long enough to allow us to assess differences in predictors of late atherosclerosis and graft failure, the follow-up periods were too short to directly assess graft failure itself. Nevertheless, at a 4.5-year angiographic follow-up of the VEST I trial, no difference in terms of graft failure was found [60]. Longer angiographic and clinical follow-up of randomized studies is needed to clarify the evidence about the benefit of this device.

### 4.3. Endoscopic vs. Open Harvesting

Endoscopic vein harvesting (EVH) consists of the dissection and the harvest of the graft, videoscopically guided, through small skin incisions. This method is thought to reduce the rate of wound-related complications [61]. Despite the potential benefit of this technique, in 2009, a subgroup analysis of the PREVENT trial demonstrated that EVH was associated with a significantly higher rate of failure compared to open harvesting at a 12- to 18-month follow-up. At the 3-year follow-up, EVH was also associated with higher rates of all-cause mortality, myocardial infarction, and the need for repeat revascularization [62]. Another subgroup analysis of the ROOBY trial confirmed the previous findings, showing a significantly lower patency rate and higher repeat revascularization rate for EVH at 1-year follow-up [63]. Despite these results, it must be noted that these are subgroup analyses of RCTs that aimed to assess different questions and, therefore, were not powered to compare graft failure in EVH with conventional open vein graft harvesting. Real-world large studies demonstrated different results. A large retrospective study performed by Dacey et al. found EVH to be non-inferior in terms of mortality and need for repeat revascularization compared to open harvesting in a population of 8542 patients [64]. Furthermore, an observational study of 235,394 Medicare patients found that the use of EVH is not associated with increased mortality [65]. In 2013, a meta-analysis of 267,525 patients showed that EVH is a safe technique with demonstrated benefits in terms of postoperative pain and wound infection. No difference in postoperative MI, mid-term MI, mid-term mortality, recurrence of angina, or repeat revascularization were found [66]. The REGROUP trial is the only large, multicenter RCT adequately powered to assess outcomes of EVH versus open vein harvesting, and in 2019, Zenati et al. published the first results. They did not find a significant difference between open vein-graft harvesting and endoscopic vein-graft harvesting in the risk of major adverse cardiac events, including death from any cause, nonfatal myocardial infarction, or repeat revascularization at a median follow-up of 2.78 years [67]. These results were confirmed at a 4.7-year follow-up [68]. Lack of imaging evaluation of the grafts, use of only experienced endoscopic harvesters, and exclusion of the off-pump CABG and the no-touch harvesting technique were important limitations of the study, which limited the applications of the findings. In view of the previous evidence, the 2018 European Society of Cardiology/European Association for Cardio-Thoracic Surgery guidelines recommended that endoscopic harvesting of SVGs should be performed by experienced surgeons [5]. Most of the EVH procedures used a skeletonized technique despite previously discussed evidence showing a strong superiority of the NT-SVG. In view of the advantage of the EVH in terms of wound healing and the patency advantage of the no-touch technique, few attempts have been made to merge the two techniques to take the best of both [69]. The high technical complexity of this procedure, which increases the operative time, has so far limited its application. 

### 4.4. Vein Preservation

As the vein graft is a living tissue, the ideal storage solution should preserve and restore endothelial function after harvesting. Traditionally, grafts have been stored in 0.9% normal saline mixed with heparin. However, this solution is acidic and hypertonic and may contribute to damage to the endothelium and, subsequently, the promotion of neointimal hyperplasia [70]. An alternative solution is represented by autologous whole blood, which is always available intraoperatively and is an alkaline solution. Studies that compared this with normal saline have found little evidence of its advantages with unclear benefits [71,72]. Another option is to use a buffered solution, and a retrospective analysis of the PREVENT IV trial demonstrated that patients undergoing CABG whose vein grafts were preserved in a buffered saline solution had lower vein graft failure rates and a trend towards better long-term clinical outcomes compared with patients whose grafts were preserved in saline- or blood-based solutions [73]. One of the buffered solutions under investigation is Duragraft, which is a pH-balanced physiological salt solution containing glutathione, L-ascorbic acid, and L-arginine. A recent RCT compared Duragraft versus normal saline and demonstrated a favorable effect of the Duragraft on wall thickness at 12 months, particularly in the proximal segment, compared to the control solution [74]. Further studies and a longer follow-up are warranted, and the European, multicentric, prospective VASC registry will assess the 5-year clinical outcomes of Duragraft compared to normal saline. 

### 4.5. Anastomosis and Graft Configuration

Where and how to anastomose a graft seems to be fundamental to its long-term patency, and this is largely dependent on the caliber of the target vessel and its capacity to provide an adequate runoff [22]. There is evidence that an arterial-venous composite graft provides advantages compared to aorto-venous proximal anastomosis. In the first setting, not only is the SVG subjected to a dampened pressure compared to the latter, but also, when the proximal anastomosis is connected to an internal mammary artery, the SVG experiences the beneficial effects of the vasodilatory, antithrombotic, and antiatherosclerosis mediators from the IMA [75]. SAVE-RITA was an RCT that compared SVG and RIMA as Y-composite grafts attached to the LIMA in situ, and it showed that the SVG was not inferior to the RIMA in terms of angiographic patency at 1-year follow-up [76]. A further improvement in the follow-up patency rate has been shown with the use of NT-SVG as a composite graft arising from the internal thoracic mammary [77]. These data suggest the need for further studies with longer follow-ups to definitively assess the benefit of arterial-venous proximal anastomosis in CABG, even though the current evidence and the pathophysiology warrant a more extensive adoption of this technique.

## 5. Pharmacological Treatment

Medications given after CABG have the main purpose of preventing graft stenosis and, by targeting the atherosclerotic risk factors, should reduce the rate of long-term failure. The mainstay of post-CABG therapy is usually a single (SAPT) or dual anti-platelet therapy (DAPT) plus a statin. The American Heart Association (AHA) recommends the use of anti-platelet and statin for all patients undergoing CABG [78]. Aspirin should initially be administered within 6 h after surgery and continued indefinitely. The use of dual anti-platelet therapy is strongly suggested in off-pump CABG, but no clear evidence is available for on-pump CABG. The reason why DAPT should be used in off-pump CABG is that, despite the avoidance of extracorporeal circulation, this procedure induces a transient state of hypercoagulability and aspirin resistance in the early postoperative period [79]. While the evidence for the benefits of aspirin has been consolidated for decades, recent studies have been undertaken to assess the addition of a second anti-platelet agent and which should be the agent of choice. A recent meta-analysis showed an important absolute benefit of adding ticagrelor or clopidogrel to aspirin to prevent saphenous vein graft failure. However, a sensitivity analysis with only on-pump CABG showed a significant advantage only for ticagrelor and not for clopidogrel [80]. The DACAB trial was a multicenter RCT that compared the effect of ticagrelor and aspirin versus ticagrelor alone versus aspirin alone on saphenous vein graft patency at 1 year after CABG. It was shown that ticagrelor and aspirin together significantly increased graft patency after 1 year compared with aspirin alone. There was no significant difference between ticagrelor alone and aspirin alone [81]. Conversely, the POPular CABG, comparing aspirin and ticagrelor with aspirin alone, found that the addition of ticagrelor to standard aspirin did not reduce the SVG occlusion rate at 1 year after CABG [82]. In the DACAB trial, 75% of patients underwent off-pump coronary surgery, while in the POPular CABG trial, more than 90% of patients were operated on-pump. This further confirmed the benefit of DAPT following off-pump CABG but not necessarily following on-pump CABG. 

Hyperlipidemia is a major contributor to atherosclerosis and, also, to vein graft disease. The AHA guidelines suggest aggressive control of cholesterol levels before and after surgery [78], and the mainstay medication to lower lipid levels is statins. In particular, it is low-density lipids (LDL) levels that are involved in the atherosclerotic process and, therefore, have become the focus of studies aiming to evaluate the relationship between cholesterol and vein graft failure. The Post Coronary Artery Bypass Graft Trial investigators showed that aggressive lowering of LDL cholesterol levels to below 100 mg per deciliter reduced the progression of atherosclerosis in grafts, and analysis from the CASCADE trial confirmed that LDL levels less than 100 mg/dL were independently associated with improved graft patency [36,83]. Furthermore, the latter showed no further improvement in graft patency with LDL reduction to below 70 mg/dL. 

Overall, the impact of optimal medical therapy (OMT) on the clinical outcomes of CABG seems to be fundamental. A sub-analysis of the SYNTAX trial found that medication status at 5 years had a significant impact on 10-year mortality, and patients on OMT with guideline-recommended pharmacologic therapy at 5 years had a survival benefit [84].

## 6. Treatment of SVG Failure

Despite all the previously described precautions, the failure of grafts after CABG remains a problem for a notable number of patients. For some patients, vein graft occlusion develops insidiously, but when it becomes clinically relevant, it must be addressed properly and promptly. In a patient with stable disease, the revascularization decision should be guided by the severity of symptoms and non-invasive evidence of ischemia in the myocardial region supplied by the graft [79]. Wolny et colleagues proposed an algorithm for the treatment of SVG failure, and the first step was to assess if acute coronary syndrome, persistent symptoms, significant unprotected left main or proximal LAD lesion, or ischemia in an SVG-supplied region were present concomitantly to SVG significant stenosis or occlusion. If not, medical management was suggested, as the isolated evidence of SVG failure does not indicate any treatment. On the other hand, if one of these concomitant factors were present, they suggested evaluating re-doing CABG as the first choice and PCI of the native vessel as the second choice. A recent meta-analysis showed that in patients with prior CABG, PCI is associated with better operative outcomes, but re-doing CABG is associated with better survival and freedom from repeat revascularization at follow-up [85]. Re-doing CABG should, therefore, be considered in young patients with relatively low operative risk, especially when the internal mammary artery has not been already grafted. Brilakis et al., in an American cohort of more than 11,000 patients undergoing PCI after CABG, showed that compared with native coronary PCI, bypass graft PCI was significantly associated with higher incidences of short- and long-term major adverse events, including more than double the rate of in-hospital mortality [86]. Only when native vessel PCI is not possible should PCI to SVG be performed.

## 7. Conclusions and Future Directions

In conclusion, SVG remains the most used graft in CABG after the LIMA; despite this, its patency has been described as inferior to other arterial grafts. In this review, we have described many technologies and techniques that could significantly improve the clinical and angiographic outcomes of vein conduits. More trials need to be conducted to reinforce and strengthen the evidence available; also, trials comparing PCI vs. CABG should consider the gold standard for both strategies. In trials considered the most advanced and new stent technology for the PCI, they should select CABG performed according to the best current medical practice. Currently, it is the use of multi-arterial grafts or the use of vein grafts harvested and anastomosed following the techniques and using the technologies that are proven to improve their patency.

## Data Availability

Not applicable.

## Abbreviations

CABG	coronary artery bypass grafting
CCTA	coronary computed tomography angiography
C-SVG	conventionally harvested—saphenous vein graft
DAPT	dual anti-platelet therapy
EVH	endoscopic vein harvesting
GEA	gastro-epiploic artery
IMA	internal mammary artery
LAD	left anterior descending
LDL	low-density lipids
LIMA	left internal mammary artery
NT-SVG	“no-touch” harvested—saphenous vein graft
OMT	optimal medical therapy
PCI	percutaneous coronary intervention
RA	radial artery
RCT	randomized controlled trial
RIMA	right internal mammary artery
SAPT	single anti-platelet therapy
SVG	saphenous vein graft
